# Best Practices to overcome challenges and barriers during the implementation of a data space for the energy domain: An experience report

**DOI:** 10.1016/j.dib.2025.111838

**Published:** 2025-06-25

**Authors:** Sebastian Copei, Linda Rülicke

**Affiliations:** Inovationfield Digital Ecosystems, Fraunhofer IEE, Joseph-Beuys-Straße 8, Kassel, 34121, Hessen, Germany

**Keywords:** Data space, Energy domain, Architecture, Challenges, Barriers

## Abstract

With multiple national and international data space projects, the drive of this technology may change data-sharing processes disruptively. With the promise of sovereign, secured, and trustful data exchange, a data space seems perfect for closing the gap in inter-company communication through various domains. The energy domain in Germany is also currently in the digital transformation stage. With complex processes across multiple companies in regulated and unregulated scopes, the rigorous fulfillment of the General Data Protection Regulation (GDPR) and national security requirements is mandatory. However, data spaces seem to be a possible technology that can fulfill the requirements while allowing flexibility in implementing all needed processes. With this paper, we want to share our experiences with the implementation of a data space in the German energy domain. In addition to the general challenges and barriers that occur while creating a data space, we will outline specific challenges and barriers from the energy domain in Germany. Furthermore, we will present our solutions to overcome some of these challenges and barriers and collect them as Best Practices that can be adopted from other data space initiatives.

## 1 Introduction

Exchanging data in a system-of-systems environment presents numerous obstacles across various levels. Companies often face a fundamental dilemma: while they need to share data, they are hesitant to give away ownership and instead prefer to keep the data closed up. With selective automated usage rights management, provided by data spaces, this dilemma could be circumnavigated. Additionally, the technical integration of many heterogeneous systems is both time-intensive and complex. The emerging technology of data spaces offers concepts and ideas to address these issues. However, data spaces themselves introduce a unique set of challenges that must be carefully navigated. In this paper, the authors explore these challenges from two perspectives: a general overview applicable across domains and a focused analysis specific to the energy sector in Germany.

A major goal of this paper is to prevent future projects from repeatedly encountering the same issues by providing a comprehensive analysis of the barriers and challenges experienced across various initiatives. For researchers, practitioners, and policy makers, understanding these challenges is essential not only for designing robust, secure, and efficient data-sharing infrastructures but also for informing policy interventions. Additionally for some challenges Best Practices are provided, that helped mitigated the observed challenge in one of the data space projects the authors engaged in.

Our work is structured around three key questions:

i) What are the general barriers and challenges encountered during the implementation of data spaces?
ii) What additional barriers and challenges are specific to the energy domain in Germany?
iii) What could be possible Best Practices to mitigate or react to the listed challenges and barriers?

The foundational point for this study is the work of Hellmeier et al. [1]., which explores the concept of data sovereignty and its practical implementation in cross-company data-sharing ecosystems. Their study, based on expert interviews, outlines key requirements and challenges, highlighting the need for mechanisms that allow secure data exchange while preserving data sovereignty. We expanded this initial framework through additional literature reviews on barriers, challenges, and Best Practices related to data spaces in general and within the energy domain.

Chapter 2 provides an explanation of the concept of data spaces and delves into the specific challenges faced by the energy domain.

Chapter 3 explains the used methodology in more detail and chapter 4 provides the results of the research work. In 4.1 barriers and in 4.2 challenges from a general perspective are identified. Likewise, in 4.3 and 4.4 the barriers and challenges from the energy domain perspective are identified. The authors enriched the literature research with the experience through our recent work in different research projects, such as “energy data-X” [2], "Enershare" [3], “FAIRWinDS” [4] and "Test field environment within the 'Use Case Energy' for the establishment of the German Federal Government's Data Institute" [5], which span both national and international scopes. The "energy data-X" project aims to establish an energy data space tailored to the specific requirements of the German energy sector, for which we are responsible for designing the overall data space architecture. The "Enershare" project seeks to develop a European-scale energy data space, where we contribute by implementing a connector that facilitates a federated learning approach for anomaly detection. The "FAIRWinDS" project focuses on creating a data space for wind energy data, examining key aspects such as data findability, accessibility, interpretability, and reusability. In this initiative, we have implemented distributed calculations for data aggregation through connectors. Lastly, the "test field environment" project also endeavors to establish an energy data space in Germany, with an emphasis on the processes as well as the challenges and barriers associated with data space implementation. As project lead, we developed the overarching concept and architecture of this data space.

In chapter 4.5, we formulated Best Practices derived from our experience in the named research projects that could mitigate the identified barriers and challenges.

Finally, chapter 5 gives a short conclusion and outlook.

## 2 Background

### 2.1 Concept data space

In early conceptions of data spaces, as presented by Halevy et al. in 2006, the focus was on managing large, diverse sets of data sources without requiring full semantic integration [6]. Their approach, termed DataSpace Support Platforms, emphasized the capability to connect and interact with heterogeneous data sources within a flexible framework that allowed for incremental integration. This concept differed from traditional data integration models to a “data coexistence” model, where useful services could be provided across data sources despite partial or absent integration efforts. The DataSpace Support Platforms approach tackled key challenges in data management, including data discovery, search, and lineage tracking, and it enabled the system to offer basic functionalities like keyword search without necessitating comprehensive integration. Data lineage was essential in this framework, providing traceability across loosely connected data sources and enhancing trust in the data’s origin and accuracy.

Nowadays, a data space is defined by the Data Space Support Centre (DSSC) as follows:

“A distributed system defined by a governance framework that enables secure and trustworthy data transactions between participants while supporting trust and data sovereignty. A data space is implemented by one or more infrastructures and enables one or more use cases” [7].

**Fig. 1:**
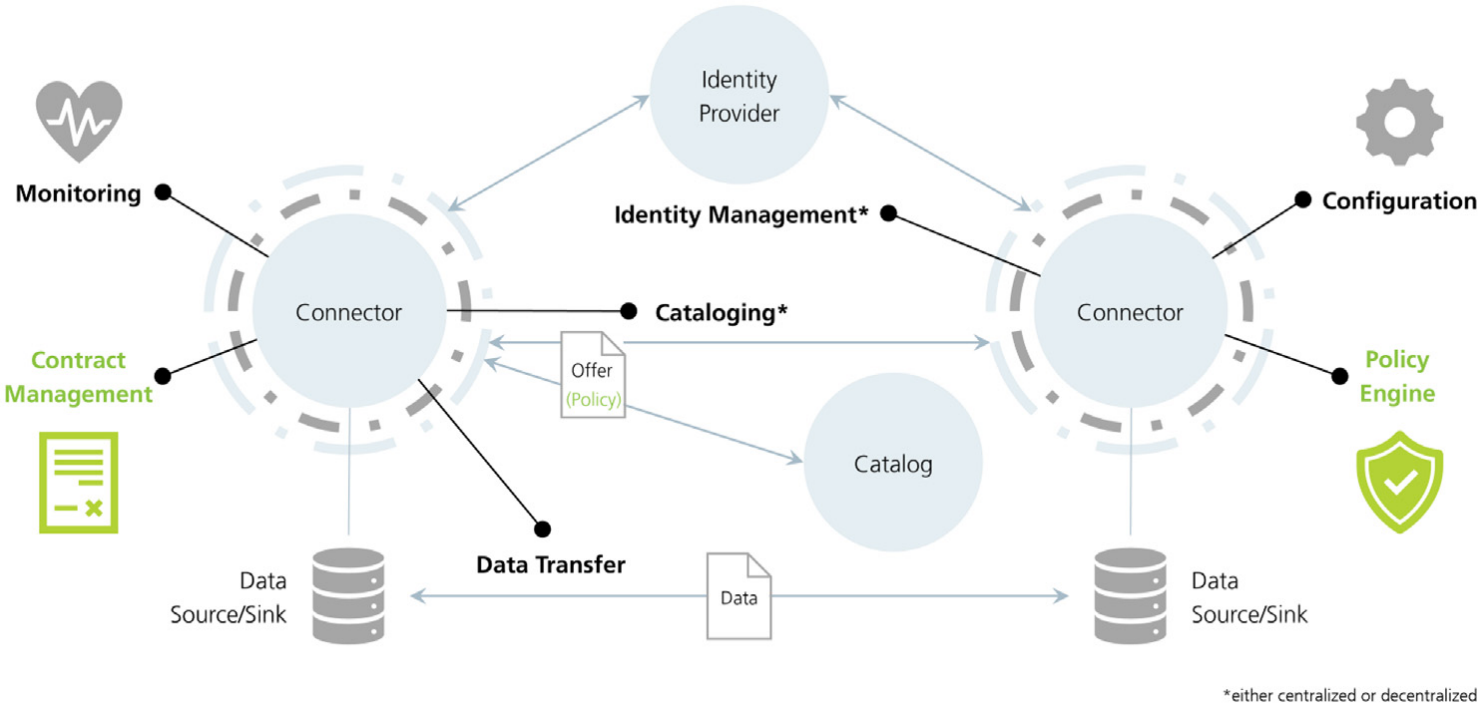
Overview of data space components [9].

The DSSC also provides a blueprint covering organizational, legal, governance, business, and technical perspectives on the data space, giving a good introduction to the general world of data spaces and their terms [8]. Furthermore, the International Data Space Association (IDSA) supports the DSSC's mission by providing a reference architecture. Fig. 1 depicts the components of a data space and their relations [9]. Interactions within a data space are facilitated by Connectors, which serve as technical interfaces integrated into each participant's system. Every participant must operate at least one Connector to interact within the data space. To participate, entities must first claim their identity from a trusted Identity Provider, which verifies and validates this identity. This validation process allows the Identity Provider to grant authorization and assign data access rights. Participants acting as data providers can make data available by creating detailed offers, which adhere to predefined standards and can specify the permitted usage of the shared data. These offers are accessible to data consumers either directly through the data provider's Connector or via a shared Catalog within the data space. Consumers can explore the Catalog, select offers, and initiate a data transaction by sending a contract request directly to the data provider. Once mutual consent is established through a contractual agreement, the data transfer process is enabled, allowing data to flow securely from the data provider's source to the data consumer's sink, governed by the agreed terms of use.

### 2.2 Domain specific challenges of the energy sector

Data availability is crucial in the energy domain, yet the necessary data is often inaccessible. For instance, planning a city's heating network requires a wide range of data, which is frequently unavailable to the planners, hindering effective decision-making and implementation. Major challenges include to get the data from chimney sweeper or to map it to the houses in accordance with the General Data Protection Regulation (GDPR) [10].

Additionally, all providers of digital twin solutions in the energy sector rely on data that is accessible in a standardized format. Every customization introduces additional adaptation work, which not only increases complexity but also further reduces the already limited profit margins in this domain. Standardization is therefore essential to ensure efficiency and sustainability [11].

The challenge of data availability extends beyond the local level, as pan-European data exchange and sharing are equally critical. Enabling cross-border electricity markets within the European Union requires seamless access to and sharing of data, which is essential for fostering integration, efficiency, and collaboration across member states [12].

So, the hunger for easy consumable data in the energy domain is immense. But existing standards or regulations do not make it possible to get the needed data in an easy manner. In the German energy sector for example, data exchange for regulated transactions is governed by the standardized process of Market Communication (MaKo). This information flow is built around the interactions between market participants, each uniquely identified by a Market Partner ID. As of now, over 4,500 organizations with more than 10,000 market partners are registered with the German Association of Energy and Water Industries (BDEW), which oversees the assignment of these market codes [13].

Similar to the data space approach, the MaKo operates on a peer-to-peer communication principle, where messages are exchanged directly between the parties involved. Despite the structured framework, the system faces significant challenges in practice. The implementation and handling of MaKo processes vary widely among market participants, leading to inconsistent adoption of certain processes and support for specific messages. Data interpretations can differ, errors are treated and reported inconsistently, and cross-partner master data reconciliation remains particularly problematic.

These issues are likely to intensify as the scope of MaKo expands. The last four years have seen significant growth in market processes and communications, particularly with the increased adoption of intelligent metering systems in large-scale daily operations [14]. This shift is expected to bring even higher manual effort and associated costs.

In conclusion, data availability is a critical issue in the energy sector, spanning from local to pan-European operations. On a local scale, planners often struggle to access the necessary data. Digital twin providers also face significant hurdles due to the lack of standardized data formats. On a broader level, pan-European data exchange is essential for strengthening cross-border electricity markets and fostering collaboration within the EU. However existing standards like the German MaKo reveal considerable inconsistencies, including variable adoption of processes, differing data interpretations, and challenges in master data reconciliation. These issues are exacerbated by the growing complexity of market processes, driven by the integration of intelligent metering systems. As the energy sector evolves, addressing these data availability and interoperability challenges will be critical to achieving efficiency and sustainability across local and international domains.

## 3 Methodology

To explore the challenges, barriers, and Best Practices associated with the implementation of data spaces, we adopted a lean literature review, which we combined with the author’s experience in multiple data space implementation projects. Rather than aiming for a comprehensive meta-analysis, our objective was to provide an experience-based account that is grounded in selected literature. The goal is to share practical insights, supported by relevant academic sources, rather than to exhaustively map or synthesize the entire body of existing research.

As outlined in the introduction, our analysis began with the foundational work of Hellmeier et al. [1], which examines the concept of data sovereignty in cross-organizational data sharing. Their categorization of challenges into organizational and technical domains provided a valuable framework for structuring the findings of this study. To capture a broader spectrum of issues discussed in the literature, we extended this framework by introducing a third category—socio-economic—which reflects challenges such as user engagement, trust, and incentive mechanisms.

In the next step, relevant literature was identified through a targeted screening process. As a first step search strings to address the key research questions were formulated. These search strings were applied across multiple scientific databases.

The following databases were consulted:

• ACM Digital Library (https://dl.acm.org/, accessed on 07 April 2025)
• Oxford Academic (https://academic.oup.com/, accessed on 07 April 2025)
• Springer (https://link.springer.com, accessed on 07 April 2025)

The search strings used in each database are summarized in the table below:

**Table:** 

Database	Search String
ACM Digital Library	*[[Title: "data space"] OR [Title: "data spaces"]] AND [[Title: "barrier"] OR [Title: "barriers"] OR [Title: "challenge"] OR [Title: "challenges"] OR [Title: "best practice"] OR [Title: "Best Practices"]]* *OR* *Title:("energy data space" OR "energy data spaces") AND Abstract:("barrier" OR "barriers" OR "challenge" OR "challenges" OR "Best Practice" OR "Best Practices")*
Oxford Academic	*("data space" OR "data spaces") AND ("challenge" OR "challenges" OR "barriers" OR "barrier" OR "Best Practice" OR "Best Practices"))*
Springer	*("data space" OR "data spaces") AND ("challenge" OR "challenges" OR "barriers" OR "barrier" OR "Best Practice" OR "Best Practices"))*

To ensure the relevance and quality of the retrieved literature, we applied the following inclusion criteria:

• Publication period: 2015–2025
• Document type: Peer-reviewed journal articles and conference proceedings
• Language: English and German
• Availability: Open access

As a result the following articles were found:

**Table:** 

Database	Search String
ACM Digital Library	[15*,*^16^]
Oxford Academic	[17]
Springer	[18,19]

Furthermore, one particularly relevant publication was added to the result set although it is not indexed in the selected academic databases: the Energy Data Space policy paper published by the Publications Office of the European Union. This policy paper provides valuable insights into both the technical and organizational aspects of energy data spaces and complements the peer-reviewed literature with a practitioner-oriented perspective on regulation, governance, and standardization challenges in the European energy context [20].

After quality checking [18] was removed as it is not describing any challenges, barriers or Best Practices in the development of data space in specific but is describing mainly the proposal of the European commission to establish a European Health Data Space [21].

## 4 Results

This chapter presents the key findings of the study, focusing on the challenges and barriers encountered in the implementation of data spaces and the corresponding Best Practices that address them.

Based on the identified literature in the above-mentioned chapter, this chapter provides a list of challenges, barriers and Best Practices, the authors experienced as well during the project work. In the identified literature partially additional challenges were listed, which we cannot relate to or do not agree in the importance for a practical implementation. Those challenges are not included. Consequently, we did not add "own observation" as a source for challenges or barriers already documented in the literature but only for those not previously mentioned.

In contrast to the investigated literature, we differentiate between a challenge and a barrier. From the general definition of a challenge [22] and barrier [23], we derive the following definitions. A challenge is a detected hurdle but is not a deal breaker for a data space project. A barrier, on the other hand, can be crucial. Without an appropriate mitigation, a barrier has the potential to not only block a data space project but also completely prevent its implementation.

The following four subchapters provide a structured overview of the identified challenges and barriers related to the implementation of data spaces. A summary of all findings can be found in Table 1.

**Table 1:** Overview of all challenges and barriers.

Domain	#	Type	Name	Category	Source
General	B1	Barrier	Dependency on small Open-Source Community	Organizational	Self-observation
General	B2	Barrier	Identity Management	Technical	[20]
General	B3	Barrier	Interoperability between software stacks	Technical	[1,15]
General	B4	Barrier	Access & Usage Control	Technical	[1,17,19]
General	C1	Challenge	All participants need to trust the data space operator	Organizational	[20]
General	C2	Challenge	Limited resources	Organizational	[1,19]
General	C3	Challenge	Paper contracts remain	Organizational	[1]
General	C4	Challenge	Business & Economics	Organizational	[1]
General	C5	Challenge	Consent Management	Organizational	[17]
General	C6	Challenge	Concerns about revealing proprietary know-how	Socio-economical	[1,19,20]
General	C7	Challenge	Data valorization	Socio-economical	[15,19,20]
General	C8	Challenge	Comfort	Socio-economical	[1]
General	C9	Challenge	Transfer frequency	Technical	[19]
General	C10	Challenge	Business Logic stays in the backend	Technical	Self-observation
General	C11	Challenge	Few operational examples	Technical	Self-observation
Energy	B5	Barrier	Participation barriers	Organizational	[17]
Energy	C12	Challenge	Different country specific standards	Organizational	[20]
Energy	C13	Challenge	Requirements for critical infrastructure	Organizational	Self-observation
Energy	C14	Challenge	Harmonization of data models, ontologies and architectures	Technical	[15,16,20]
Energy	C15	Challenge	Accessibility of data	Technical	[20]
Energy	C16	Challenge	Personal Data	Technical	[20]
Energy	C17	Challenge	Reusage of established identity methods	Technical	Self-observation

To facilitate cross-referencing throughout the chapter, each challenge and barrier is assigned a unique ID, which is used in the subsequent text and descriptions.

### 4.1 General barrier

**Organizational**

One barrier concerning the practical implementation of data spaces is the high dependency on a small open-source community (B1). Currently, the open-source developer community in the field of data spaces is relatively small. This results in insufficient or nonexistent documentation and, therefore, very high adoption hurdles for developers. Especially if the software is seen as a ready-to-use package, adoption may not be possible if there are no developer capacities who are able to make needed adjustments or roll out the software. Most of the implementation projects of data spaces take the available open-source software and use it for their use case. A lack of well documented or out-dated open-source code would increase the costs of the implementation of a data space further as basis infrastructure components need to be updated by each project. Due to the significant impact on possible participants in a data space, the authors see this not only as a challenge, but as a barrier.

**Technical**

Identity management (B2) represents a fundamental component of every data space architecture, as it must ensure that each participant is reliably verified and authenticated. Efficient and secure identity management is therefore essential for establishing trust among participants and safeguarding data exchange. Beyond this foundational role, identity management is also critical for achieving interoperability between different data spaces. To enable cross-domain and cross-border collaboration, data spaces must be able to mutually recognize and accept the trust levels and credentials issued by other systems. As a best practice to address this challenge, the Electronic Identification and Authentication Services (eIDAS) regulation, along with electronic identification schemes (eIDs), provides a promising foundation. This European framework offers a standardized and trustworthy approach to identity verification across all EU Member States, making it a suitable candidate for connecting and harmonizing identity management across multiple data spaces within the EU [20].

Another barrier is the lack of interoperability between different software stacks (B3) used in existing data space implementations. As highlighted in the Data Connector Report [24], the growing number of incompatible technologies leads to the creation of new data silos, which goes against the core goal of building an integrated and collaborative data-sharing ecosystem. Efforts such as the development of a standardized data space protocol [25] are essential to overcome this fragmentation by enabling interoperability between various implementations. The Data Space Protocol is not a standard yet, but aims to submit to ISO standard [26].

A fourth barrier lies in access and usage control (B4). While some access control solutions are already in place, significant gaps remain—particularly in tailoring access rights to organizational roles. Usage control, which governs how data may be used after access is granted, presents even greater complexity. Full automation of usage policy enforcement is considered unrealistic by some stakeholders, as technical enforcement mechanisms are difficult to implement on the data-consuming side. As a result, effective and reliable policy enforcement remains a key challenge in ensuring compliance and maintaining trust among data space participants [1,17].

### 4.2 General challenge

**Organizational**

A fundamental organizational challenge (C1) in the implementation of data spaces is the central role of the Data Space Operator. As the entity responsible for managing core infrastructure components and ensuring reliable operation, the operator have the trust of all participants within the ecosystem. This trust originates from factors such as transparency, neutrality, and the responsible handling of sensitive data. In addition to trust, the operator must possess sufficient financial stability and access to skilled IT resources to operate and maintain the infrastructure effectively. To address this challenge, it is essential to define the operator model early in the design phase of the data space. Different options for legal structure, governance, and business models should be evaluated carefully. This includes assessing the risk of dependency or lock-in effects, which could limit flexibility and long-term sustainability [20].

The implementation of data spaces introduces a high level of technical complexity (C2), which requires the involvement of experienced and highly skilled IT professionals. However, such experts are often in high demand and frequently engaged in other strategic projects, leading to internal competition for their availability. This scarcity of qualified personnel can result in delays in the development and rollout of a data space. As a best practice, organizations can consider using managed services offered by specialized IT providers to simplify the initial setup and operation of a data space. This approach enables a faster entry into sovereign data sharing, although it is important to carefully weigh the trade-off between ease of use and the potential loss of control over data and processes. In the longer term, the development and distribution of user-friendly documentation or contributions to open-source repositories can significantly reduce the complexity for new participants, thereby supporting broader adoption and reducing reliance on scarce internal resources [1].

Despite the ability of data spaces to automate contract negotiation, traditional paper contracts prepared by legal departments are likely to remain in use (C3). Legal and regulatory requirements, as well as organizational preferences, often necessitate formal agreements beyond digital mechanisms [1].

From a business and economic perspective, effective data management in data spaces requires additional technical effort, which translates into increased costs (C4). Although many initial solutions are available as free and open-source software, their customization, integration, and maintenance still demand significant resources. To justify these investments, it is crucial to communicate the expected benefits of the data space solution clearly and early in the process [1].

Participation in a data space does not grant automatic access to all available data (C5). Each use of a data asset requires explicit consent from the data owner, making active consent management essential. This ensures that data is only accessed when permission has been clearly granted, upholding key principles of data sovereignty and privacy [17].

**Socio-economical**

Companies are often hesitant to share data due to concerns about revealing proprietary know-how (C6), which poses a significant challenge to building the trust required for active participation in data spaces. As a Best Practice, clearly communicating both the possibilities and, more importantly, the limitations of data sovereignty can increase transparency and enable more accurate risk assessments. This, in turn, supports informed decision-making and fosters greater confidence among potential participants [1,20].

A key challenge in data spaces is to kick-start the process of data exchange and make data valorization possible (C7). For data sharing to gain momentum, it is essential to establish effective incentive mechanisms and develop efficient data marketplaces that clearly demonstrate the value of participation. Data providers, in particular, must see tangible benefits in offering their data. As a Best Practice, such marketplaces should be designed using a use case-first approach. By focusing on specific, practical applications from the outset, the advantages of joining the data space become more visible, helping to initiate data flows and unlock the potential for data-driven value creation [15,19,20].

Enhancing data sovereignty within a data space requires the implementation of additional organizational and technical safeguards. While these measures are essential for ensuring secure and controlled data sharing, they often lead to reduced comfort (C8) and increased complexity in day-to-day operations. This causes the need to change business processes in order to match them with the data sovereignty vision. This change impact should be made as usable as possible to the final user. As a Best Practice, it is important to design not only the core technology but also the surrounding tools with a strong user-centered focus. This helps maintain usability and encourages adoption, even when stricter controls and processes are in place [1].

**Technical**

A technical challenge in decentralized data space environments is the requirement that data remains with its original owner. This means that every time a data consumer needs access, the data must be retrieved directly from the source. As a result, the overall volume of data transmitted across the network increases (C9). This situation places new demands on system architecture. It becomes necessary to ensure that real-time operations can scale effectively, even when data is stored across multiple, potentially distant, locations. At the same time, handling continuous data streams without intermediate storage requires efficient mechanisms for processing data as it flows [19].

In the Blueprint 1.5 of the Data Space Support Center the following benefit of a data space is stated:

*“The business value of having a data space is that developers of data driven applications don’t need to develop many interfaces that differ per data set (e.g. by standardizing the interfaces or connectors)”* [27].

In the energy domain, data exchange is for example governed by established and standardized protocols. While this standardization ensures a common understanding of data formats and communication flows, it also creates a significant rigidity: any modification to the standard—for instance, the addition of a new data field—requires all dependent processes and applications to be updated accordingly. This leads to a considerable amount of effort, particularly in adapting backend systems that are tightly coupled to the existing data structure. The energy domain only serves as an example, the challenge that the business logic remains in the backend (C10) exists for all domains. There is a common expectation that the adoption of a data space architecture would alleviate this burden by introducing greater flexibility and abstraction. However, this expectation often does not hold in practice. While the data space can indeed provide a uniform and standardized interface through the connector, the internal systems of each participant still need to be capable of processing the full and potentially updated data payload. As a result, the underlying backend infrastructure must be adapted in parallel to any changes in the data format—an effort that remains largely unaffected by the introduction of data space technology. Consequently, the core technical challenge of maintaining compatibility across evolving systems persists. The data space does not abstract away this layer of complexity; rather, it shifts the problem into a more federated context, where coordinated adaptation becomes even more essential.

An additional self-observed challenge is the lack of suitable documentation and replication descriptions (C11) provided by ongoing or finished data space projects. This circumstance inevitably leads to the problem of new data space projects needing to start from scratch despite the enormous amount of preparatory work.

### 4.3 Energy domain specific barrier

A notable participation barrier (B5) in data spaces is the restricted access to data, which is typically limited to companies operating within the energy sector. For digital service providers that fall outside this classification, direct participation as independent entities is often not permitted [17].

In the energy domain the only viable path into the data space may be through collaboration with an Energy Service Provider (ESP). However, this arrangement can impose limitations on both the scope of accessible data and the level of operational flexibility, potentially reducing the attractiveness and effectiveness of participation for non-energy actors.

### 4.4 Energy domain specific challenge

**Organizational**

A major challenge in establishing a pan-European energy data space lies in the considerable differences among EU member states (C12) in terms of regulatory frameworks, legacy IT infrastructures, and established data practices. These national variations hinder interoperability and make cross-border collaboration and seamless data exchange difficult. As a Best Practice, a federated data space ecosystem is recommended. This approach envisions multiple interoperable national data spaces, each tailored to local regulations and infrastructures, but built on shared semantics and ontologies. Within this framework, the pan-European energy data space would function as a unified structure with clearly defined governance, a common architecture, and standardized components [20].

Additionally, and similar to Challenge C11, the regulatory framework governing critical infrastructure in Germany is highly restrictive (C13). In the regulated segment of the energy market, there is a significant risk that data space technologies may not fully comply with these stringent requirements. As a result, affected market participants could be excluded from participating in the data space. This is a challenge the authors experienced in their work within the different projects.

**Technical**

Achieving consistency and interoperability in the energy domain requires the alignment and integration of domain-specific ontologies (C14). However, this process is highly dependent on the particular use cases and the way individual ontologies are structured to work with one another. As a Best Practice, the focus should not be on developing entirely new standards or data models. Instead, interoperability can be effectively achieved by mapping between existing models and standards [15,16,20].

Access to smart meter data (C15) remains a significant limitation, as many EU countries have yet to complete a full nationwide rollout of these devices. Without consistent and widespread data collection, essential input for analysis and innovation is missing. As a result, the development of new, data-driven business models in the energy sector is severely constrained, hindering the potential of data spaces to support advanced services and solutions [20].

In certain use cases, the exchange of personal data within a data space is unavoidable, necessitating appropriate measures such as anonymization or pseudonymization to ensure compliance with data protection regulations (C16). As a Best Practice, the metering point operator should be integrated at the company level within the data space ecosystem. This allows for data aggregation and anonymization to occur before the data enters the data space, reducing privacy risks and processing complexity within the shared environment. Additionally, the integration of a Permission Administrator can support the automatic management of data-sharing permissions, further strengthening data governance and user control [20].

In Germany, the energy domain uses a public-key infrastructure (PKI) to create trust between market partners. However, data space trends move on to self-sovereign identities (SSI). If other identity methods are not possible, the regulated part of the energy domain in Germany is not able to join the data space due to its bonding to the PKI as an authentication method (C17).

### 4.5 Best Practices

The following chapter provides a detailed exploration of the Best Practices. These Best Practices are organized into two primary categories: general Best Practices applicable across various domains and energy-specific Best Practices tailored to address the unique challenges of the energy sector.

**General Best Practices**

Establishing data spaces effectively requires a comprehensive approach that incorporates Best Practices to address organizational, technical, and socioeconomical topics. Central to this process is the creation of a robust governance framework that provides clear definitions of roles, responsibilities, and decision-making structures. This framework must establish trust among participants. A trusted data space operator should be appointed early, with the financial resources, technical expertise, and legal authority to oversee operations and manage the underlying infrastructure. To avoid lock-in scenarios and ensure sustainability, the governance model should explore diverse legal and operational structures during the design phase, allowing for flexibility as the data space evolves [20]. Interoperability plays a critical role in the functionality of data spaces. Aligning with established standards and protocols, such as the Data Space Protocol or established domain specific standards, is essential for achieving seamless integration between diverse systems.

A user-centered design philosophy should guide the development of tools and interfaces for data spaces. Simplifying the onboarding process and making day-to-day interactions intuitive are particularly important for organizations with limited IT resources.

Companies often hesitate to share proprietary data due to concerns about misuse or loss of control. Therefore, providing clear and transparent communication about the capabilities and, critically, the limitations of data sovereignty can significantly enhance trust. This approach helps potential data space participants better understand the safeguards in place, enabling more informed risk assessments and fostering greater confidence in the data-sharing ecosystem.

Operational knowledge-sharing is a beneficial help for new adopters. Projects can build on the insights and lessons from previous initiatives by detailed documentation and the sharing of Best Practices. This collaborative approach accelerates innovation and lowers the barriers to participation for new entrants.

Making data provisioning attractive for providers is a persistent challenge that must be addressed strategically. Providers are often hesitant to share their data unless they perceive clear and tangible benefits, such as financial incentives or access to valuable insights. Without these incentives, data spaces risk limited participation. To address this, it is essential to establish a transparent marketplace where providers can realize the benefits of their contributions.

Lastly, many interoperability issues stemming from identity solutions can potentially be mitigated by leveraging emerging eIDAS technology. However, as this technology is not yet production-ready, it is important to closely monitor its development and evaluate its readiness for practical implementation in the future. Additionally, a roadmap would help to visualize the timelines and manage the uncertainties more effectively.

**Energy specific Best Practices**

In addition to the general Best Practices applicable across various domains, the energy sector presents distinct challenges and requirements that necessitate tailored approaches. In order to do so, a range of targeted Best Practices can be employed to ensure the successful implementation of an energy data space.

A critical best practice is aligning identity management systems in the energy sector with existing solutions, such as the public-key infrastructure (PKI). To support broader participation and gradual adoption of newer technologies, like SSI, data spaces should integrate with established PKI infrastructure while exploring ways to add innovative solutions. This dual compatibility increases trust among participants who rely on traditional systems while enabling forward-looking flexibility.

Harmonizing data models is another critical step toward ensuring interoperability across diverse systems in the energy domain. Established standards such as CIM and SAREF provide a robust foundation for consistent data exchange [20].

To address the complexities of varying regulatory frameworks and legacy systems across EU member states, a federated data space ecosystem offers an effective solution. This model envisions interconnected data spaces built on shared semantics and ontologies, allowing individual countries to maintain their unique regulatory and infrastructural characteristics. At the same time each country specific data space could contribute in a standardized way to a unified, pan-European energy data space [20].

Including the metering point operator at the company level in the data space ecosystem is a best practice that enhances efficiency and data security. By involving metering point operators, data aggregation and anonymization can be performed at the source, reducing the complexity and computational burden within the data space itself. This approach ensures that sensitive information is processed securely before being shared, addressing privacy concerns while maintaining data utility for analytics and decision-making. Additionally, integrating a Permission Administrator within the data space framework allows for the automated management of data-sharing permissions.

## 5 Conclusion and Outlook

This paper presented a structured overview of the challenges and barriers encountered during the implementation of data spaces, with a particular focus on the energy domain. Drawing on both literature and practical experience, we identified general and domain-specific obstacles and offered Best Practices to address them.

Among the most significant hurdles are the integration of existing identity management systems, the development of standardized data models, and the establishment of trust among participants. These aspects are foundational for enabling secure and reliable data exchange.

In the energy domain, challenges are intensified by the coexistence of legacy infrastructure, regulatory fragmentation across countries, and limited access to relevant datasets. Nevertheless, the paper has also demonstrated that several of these issues can be addressed through targeted Best Practices, including the use of established frameworks and interoperable architectures.

Looking ahead, data spaces hold transformative potential for the energy sector. By enabling secure, standardized, and sovereign data exchange, they can support innovation, enhance sustainability, and improve operational efficiency. Realizing this potential will require not only technological advancement but also a flexible and adaptable governance structure that avoids lock-in effects and ensures long-term viability. Transparent communication of the benefits of data spaces is essential to motivate data providers to participate and contribute. Building trust, supported by clear rules and incentives, is a crucial step toward encouraging broader adoption.

Future efforts should focus on developing practical implementation tools, clear operational frameworks, and replication guidelines to lower the barriers to entry. A federated approach that accommodates national specificities while ensuring interoperability at the European level appears especially promising. Continued collaboration across sectors and countries will be essential to address challenges such as standardization, regulatory alignment, and semantic integration. With these developments, data spaces can become a cornerstone of digital transformation in the energy domain.

## Ethics Statement

The authors have read and follow the ethical requirements for publication in Data in Brief and confirming that the current work does not include human subjects, animal experiments, or data collected from social media platforms.

## CRediT authorship contribution statement

**Sebastian Copei:** Conceptualization, Methodology, Writing – original draft, Writing – review & editing. **Linda Rülicke:** Investigation, Data curation, Writing – original draft, Writing – review & editing.

## Data Availability

Barriers,Challanges,BestPractices (Original data)
